# Median neuropathy after open reduction and internal fixation: when implant proximity is not the culprit

**DOI:** 10.12701/jyms.2026.43.26

**Published:** 2026-03-29

**Authors:** Berkay Yalçınkaya, Hilmi Berkan Abacıoğlu, Ahmet Furkan Çolak, Harun Kürşat Karademir, Murat Kara

**Affiliations:** Department of Physical and Rehabilitation Medicine, Hacettepe University Medical School, Ankara, Türkiye

A 32-year-old man presented with a 2-year history of numbness and tingling affecting the first three digits of the left hand, predominantly at night. His medical history was notable for multiple nondisplaced fractures of the radius and ulna sustained 2 years earlier, treated with open reduction internal fixation (ORIF) using a plate and screws. The patient reported that symptoms developed in the operated hand after surgery. Physical examination revealed weakness of the abductor pollicis brevis muscle (4/5), a positive Tinel’s sign along the median nerve, and hypoesthesia in the first three digits. Ultrasonography (US) revealed enlargement of the median nerve at the level of the pisiform bone in the short-axis view (reference value for carpal tunnel syndrome, >10 mm^2^) [1], with a cross-sectional area of 16 mm^2^ calculated at the carpal tunnel inlet by tracing the inner border of the hyperechoic epineurium. Approximately 4 cm proximal to the wrist crease [2], the median nerve was seen in contact with the fixation material; however, its fascicular morphology was preserved, and no sono-Tinel was elicited at this level (Fig. 1, Supplementary Video 1). In the transverse plane, the fascicular arrangement was not completely fixed along the course of the nerve. Subtle variations in the relative positions of the fascicles were observed, which were consistent with the intraneural plexus. The interfascicular tissue (interfascicular epineurium) appeared as an irregular hyperechoic network situated between the nerve fascicles. Overall, these findings were compatible with normal nerve histopathology [3]. Electrodiagnostic tests revealed bilateral median neuropathy at the wrist. Sensory nerve conduction studies revealed reduced conduction across the finger-wrist and palm-wrist segments bilaterally. Motor studies showed prolonged distal motor latency of the left median nerve, whereas the right-sided motor parameters were normal. Ulnar nerve studies were normal bilaterally. Overall, these findings were consistent with mild right and moderate left median neuropathy at the wrist, consistent with carpal tunnel syndrome. Conservative treatment with nocturnal wrist splinting, strengthening exercises targeting the abductor pollicis brevis muscle, and flexor tendon gliding exercises were initiated, resulting in symptomatic relief. Follow-up appointments were uneventful.

In patients undergoing ORIF, the main clinical challenge in postoperative median neuropathy lies not only in recognition but also in the precise localization of the pathological site. Implant-related median nerve irritation has been described within and proximal to the carpal tunnel. In this regard, US has emerged as a reliable and widely used imaging modality for the evaluation of upper extremity peripheral nerve disorders, particularly median nerve pathologies and carpal tunnel syndrome, offering high diagnostic accuracy together with dynamic and structural assessments of the nerve and adjacent tissues [1,2]. In the acute phase, US may show fascicular enlargement and blurred echotexture due to intrafascicular edema, with reduced delineation of the normal fascicular pattern. In some cases, an increased Doppler signal reflects reactive hyperemia. In contrast, in the chronic phase, the nerve can demonstrate increased echogenicity and loss of normal architecture because of neural fibrosis with thickened connective tissue and reduced fascicular definition, which is sometimes accompanied by nerve atrophy [3]. Volar plates and screws may cause direct nerve compression, tethering, or chronic irritation of the nerve, and radiological visualization of nerve-implant contact may intuitively suggest a causal relationship [4,5]. However, structural proximity alone does not necessarily imply functional impairment. In the present case, despite direct nerve-implant contact at the forearm level, preserved nerve morphology and the absence of a sono-Tinel sign argued against clinically relevant impingement at that site. In contrast, US evidence of median nerve enlargement in the carpal tunnel, supported by electrophysiological findings, identified the true pathological site. This distinction was clinically critical because misattributing symptoms solely to implant proximity could lead to unnecessary surgical interventions. In conclusion, the complementary use of US and electrodiagnostic studies allows the precise and clinically meaningful localization of postoperative peripheral neuropathies. While US provides high-resolution dynamic morphological assessments, electrodiagnostic studies provide functional confirmation of nerve dysfunction. When used together, they significantly enhance diagnosis and guide appropriate management [6,7].

## Figures and Tables

**Fig. 1.**
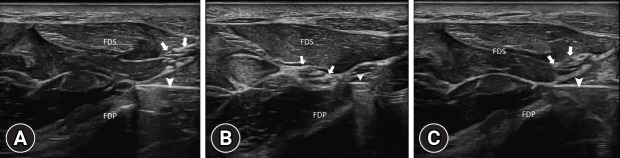
Short-axis dynamic ultrasound examination at the forearm level demonstrates the median nerve (arrows) as follows: (A) away from the screw (arrowhead), (B) near the screw, and (C) subsequently moving away from the screw. FDS, flexor digitorum superficialis; FDP, flexor digitorum profundus.

## Supplementary materials

Supplementary Video 1 can be found at https://doi.org/10.12701/jyms.2026.43.26.

Supplementary Video 1. Short-axis dynamic ultrasonographic tracking of the median nerve along the forearm demonstrates the median nerve’s (arrows) close relationship with the fixation screw (arrowhead).
